# The effect of breastfeeding education given through the teach-back method on mothers’ breastfeeding self-efficacy and breastfeeding success: a randomized controlled study

**DOI:** 10.1186/s12884-024-06601-0

**Published:** 2024-06-29

**Authors:** Elif Ocaktan Çetindemir, Eda Cangöl

**Affiliations:** 1 MSc Nurse, Susurluk State Hospital, Balıkesir, Turkey; 2 Department of Midwifery, Faculty of Health Sciences, Çanakkale Onsekiz Mart University, Çanakkale, Turkey

**Keywords:** Breastfeeding education, Breastfeeding success, Breastfeeding efficacy, Teach-back method

## Abstract

**Background:**

An individualized education using visual aids, allowing the woman to demonstrate what she has learned, and providing the opportunity for the woman to ask questions are important in terms of breastfeeding self-efficacy, breastfeeding success, and the sustainability of the education. This study is original in evaluating the effectiveness and sustainability of breastfeeding education provided through the teach-back method in terms of breastfeeding self-efficacy and success in a short period of time. Therefore, the aim of this study is to examine the impact of teach-back method on mothers’ breastfeeding self-efficacy and breastfeeding success.

**Materials and methods:**

This is a randomized controlled study. The population of this study consisted of women who gave birth in the obstetrics and gynecology department of a state hospital located in Çorlu, in the northwest region of Turkey, between March 2022 and August 2022.

The sample of this study consisted of a total of 100 postpartum women, with 50 participants in the experimental group and 50 participants in the control group, who gave birth in the obstetrics and gynecology department of Çorlu State Hospital. Computer-assisted simple randomization was employed to ensure the homogeneous distribution of the women into the experimental and control groups. The women in the experimental group received education and counseling services using the Teach-Back Method, based on the content of the prepared Breastfeeding Education Guide. The control group mothers, on the other hand, received standard breastfeeding education and counseling services. The data were collected through face-to-face interviews during the first 24 h postpartum and at the 1-month follow-up visits. In the study, the data collection tools used were a Personal Information Form, LATCH Breastfeeding Assessment and Evaluation Scale, Postpartum Breastfeeding Self-Efficacy Scale (short form), and the Teach-Back Observation Tool. In the evaluation of the research findings, the SPSS (Statistical Package for the Social Sciences) version 25.0 (IBM Corp., Armonk, NY, USA) program was used for statistical analyses. Descriptive, graphical, and statistical methods were employed to examine whether the scores obtained from each continuous variable followed a normal distribution. The Kolmogorov-Smirnov test was used to assess the normality of the scores derived from a continuous variable using statistical methods.

**Results:**

In the study, no significant difference was found in the distribution of the socio-demographic characteristics of the participants according to the study groups. In the experimental group, which received training with the tell-what-you-learned method, the mothers’ average EÖYÖ scores before the training, at the 24th hour after the training and at the 1st month after the training were 46.41 ± 11.26, respectively; It was determined to be 66.23 ± 6.94 and 67.84 ± 6.27. In the measurements made during the follow-up, it was determined that there was a significant difference in the study group’s EÖYÖ score averages (*p* < 0,001). For mothers in the experimental group, the average LATCH score of the mothers before training, 24 h after training and 1 month after training was 7.73 ± 1.81, respectively; It was determined that these values were 8.66 ± 1.61 and 9.95 ± 0.30, and there was a significant difference in the mean LATCH scores of the study group in the measurements made during the follow-up (*p* < 0.001).

**Conclusions:**

Breastfeeding education provided through the teach-back method is more effective in increasing both breastfeeding success and breastfeeding self-efficacy when compared to standard breastfeeding education.

**Trial registration:**

Iran Randomized Clinical Trial Center IRCT20220509054795N2 Date of first registration: 10/11/2022.

## Introduction

Breast milk contains all the essential nutrients that promote infant growth and development, strengthen immunity, and protect against allergic diseases such as eczema and asthma. Additionally, it helps prevent diabetes, coronary artery disease, childhood obesity, and reduces newborn illness and mortality rates [1–4]. Breast milk is an ideal nutrient with high bioavailability and digestibility [3, 4]. The benefits of breast milk extend beyond infancy and continue into adulthood [5]. In addition to all its benefits, breast milk is recognized as an interactive tool for the health of both the mother and the baby. Breast milk support until the age of 2 is considered a fundamental requirement for optimal nutrition [6]. The World Health Organization (WHO) recommends exclusive breastfeeding for infants up to 6 months of age, followed by continued breastfeeding along with complementary foods until at least 2 years of age [6–8]. However, globally, only 9% of 6-month-old infants are exclusively breastfed [9]. The rate of exclusive breastfeeding for infants during the first 6 months of their lives is 60% [10].

In Turkey, a nationwide campaign is carried out to promote exclusive breastfeeding for the first 6 months of infants’ lives [11]. Breastfeeding rates are influenced by physical factors such as pain, fatigue, and difficulties in latching the baby onto the breast. Additionally, psychosocial factors such as insufficient breast milk supply, premature or unprepared births, misconceptions about breastfeeding, women’s knowledge and self-esteem regarding breastfeeding, and pre-birth feeding decisions also affect breastfeeding rates. These factors contribute to lower breastfeeding rates both in Turkey and globally [12, 13]. Planning and implementing interventions to increase breastfeeding initiation and duration are crucial. There are both positive and negative factors that influence breastfeeding success and duration. Positive factors include the baby’s health, the mother’s higher education level, multiparity, and other socio-demographic characteristics, as well as family support. Negative factors include factors that affect the mother’s psychological state, smoking, and nipple problems [14–16]. Reducing and early cessation of breastfeeding negatively affects the health of the mother, child and society. For this reason, developing breastfeeding self-efficacy, which is one of the factors affecting breastfeeding and the sustainability of breastfeeding, is very important in breastfeeding education [17]. The process of recognizing breastfeeding self-efficacy and ensuring breastfeeding success is challenging. Education plays a significant role in enhancing a mother’s breastfeeding self-efficacy. There are various educational methods available to initiate and sustain breastfeeding. However, there is no widely accepted education method that bridges the communication gap between women and midwives/nurses and maximizes the development of self-efficacy [14, 18]. As a result, communication barriers between healthcare professionals and clients negatively impact access to quality services. Indeed, there are studies indicating that people tend to forget the information they receive regarding their health [14, 19]. The integration of the teach-back method into breastfeeding education fills communication gaps. Additionally, the method is important for promoting positive health behaviors in individuals, especially those with low health literacy, by enhancing learning and comprehension [14]. This method contributes to improving adherence to the breastfeeding process and increasing breastfeeding success, thus benefiting the health of both the mother and the baby [18].

In a study where demonstrations, practices, and informative sessions were conducted once a week, it was observed that breastfeeding and breastfeeding success increased each week [20]. In a study evaluating breastfeeding self-efficacy after education using the teach-back method, it was reported that mothers who received the education showed an increase in breastfeeding self-efficacy at postpartum weeks 1 and 6 compared to before the education [21]. As reported in the literature, both the teach-back method and other educational approaches were shown to enhance breastfeeding self-efficacy and breastfeeding success when employed in breastfeeding education. However, the results of an individualized education using visual aids, allowing the woman to demonstrate what she has learned, and providing the opportunity for the woman to ask questions are important in terms of breastfeeding self-efficacy, breastfeeding success, and the sustainability of the education. This study is original in evaluating the effectiveness and sustainability of breastfeeding education provided through the teach-back method in terms of breastfeeding self-efficacy and success in a short period of time. Therefore, the aim of this study is to examine the impact of teach-back method on mothers’ breastfeeding self-efficacy and breastfeeding success.

## Materials and methods

### Design

This is a randomized controlled study.

### Setting

The population of this study consisted of women who gave birth in the obstetrics and gynecology department of a state hospital located in Çorlu, in the northwest region of Turkey, between March 2022 and August 2022.

### Sample

**Fig. 1:**
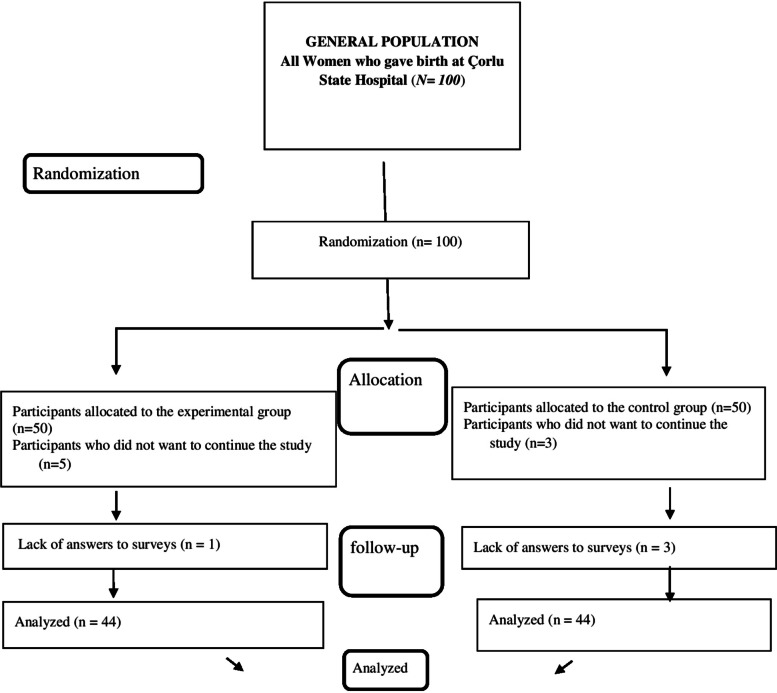
CONSORT guideline for the study

The sample size for the study was determined using power analysis with a desired effect size of d = 0.538, α = 0.05 (margin of error), and 1-β = 0.80 (power), utilizing the G-power (version 3.1) software package. A study published in 2023 was used to determine the sample size of the study [21]. Based on these criteria, a total of 88 participants, with 44 participants in each group, were initially determined. Considering possible attrition, a total of 100 participants, with 50 participants in each group, were planned to be recruited. Postpartum women who had a vaginal birth and met the inclusion criteria were included in the study after providing their consent. To ensure homogeneity in the distribution of participants between the experimental and control groups, a computer-assisted simple randomization method was employed (The CONSORT guideline is outlined in Fig. 1).

### Randomization

To ensure the homogeneous distribution of the participants into the intervention and control groups, a computer-assisted simple randomization method was employed. Utilizing functions available on the website “https://www.random.org/integer-sets”, 100 sets were generated. Each set comprised a total of 10 participants, with 5 participants from each group. These 100 sets were then represented by unique numerical identifiers. Subsequently, 10 sets to be used for randomization were randomly selected using the “RAND” function in Excel. This randomization process aimed to minimize selection bias and control variables that could potentially influence outcome parameters. Consequently, 50 participants were randomized to the intervention group and 50 to the control group.

The inclusion criteria for the sample selection were as follows:

- Being 18 years of age or older.
- Having basic literacy skills.
- Having a full-term and healthy delivery.
- Having a healthy newborn.
- No anomalies in the newborn that would hinder breastfeeding.

The exclusion criteria for the sample selection were as follows:

- Having a condition in the mother that hinders breastfeeding.
- Not being able to speak Turkish.
- Having hearing or speech impairments.

### Measurement

In the study, the data collection tools used were a Personal Information Form, LATCH Breastfeeding Assessment and Evaluation Scale, Postpartum Breastfeeding Self-Efficacy Scale (short form), and the Teach-Back Observation Tool.

### Personal information form

The personal information form consists of 19 questions. The first 14 questions are related to the socio-demographic characteristics of the postpartum women participating in the study. The last 5 questions are related to the obstetric characteristics of the postpartum women [7, 20, 22]. The personal information form was pilot tested on 10 individuals, and necessary adjustments were made before using it in the sample group.

### LATCH Breastfeeding Assessment and Evaluation Scale

The LATCH Breastfeeding Assessment and Evaluation Scale was initially developed in 1986 and is based on the scoring method similar to the APGAR score system. There are five evaluation criteria for breastfeeding. These include latching, swallowing, types of nipple, comfort level, and positioning. As the score increases, breastfeeding success improves. The Turkish reliability study of the scale was conducted by Yenal and Okumuş. The original scale has a Cronbach’s alpha value of 0.93, while the Turkish adaptation study reported a value of 0.95 [23]. This scale was used in the study to assess breastfeeding success.

### Postpartum Breastfeeding Self-Efficacy Scale (short form)

The Breastfeeding Self-Efficacy Scale was developed by Dennis in 1999 and consists of 33 items. Content validity of the BSES was based on a literature review, interviews with breast-feeding mothers, and expert judgement using a method recommended by Lynn (1986). Following a pilot test, an initial psychometric assessment was conducted with a convenience sample of 130 Canadian breast-feeding women, where questionnaires were completed during the postpartum hospitalisation and again at 6 weeks postpartum. Responses were subjected to principal components analysis with a varimax rotation, yielding the theorised subscales. Support for predictive validity was demonstrated through positive correlations between BSES scores and baby-feeding method at 6 weeks postpartum. The scale has a Cronbach’s alpha value of 0.96, indicating high internal consistency. The item-total correlations range from 0.30 to 0.70, with an average of 0.73. In 2003, a short form of the scale was developed, reducing it to 14 items. The item-total correlation of the short form is below 0.60. The short form of the scale was administered to 491 breastfeeding mothers at 1, 6, and 8 weeks postpartum, resulting in a Cronbach’s alpha value of 0.94, indicating high reliability. The scale uses a 5-point Likert-type response format, ranging from 1 = “Not at all confident” to 5 = “Very confident,” and all items are positively worded. The minimum score that can be obtained is 14, while the maximum score is 70. As the score increases, breastfeeding self-efficacy also increases [7, 24].

### Teach-back observation tool

The tool developed for the evaluation of how the teach-back method is implemented is called the Teach-Back Observation Tool. In both the initial and final stages of the research, an external observer, who is an expert in Obstetric and Women’s Health Nursing, assesses the researcher’s application of the method using the Teach-Back Observation Tool [14]. The researcher’s skill in applying the method was demonstrated with the observation tool. This tool was used in the study to demonstrate that there was no variation in research findings due to researcher-related factors.

### Study process

The data were collected through face-to-face interviews during the first 24 h postpartum and at the 1-month follow-up visits. The initial interview took place in the woman’s room at the birthing facility within the first 24 h after delivery. Prior to the education, the researcher received 70 h of breastfeeding counseling education. Pre-education data collection was conducted through face-to-face interviews using a personal information form, the LATCH Breastfeeding Assessment and Evaluation Scale, and the Postpartum Breastfeeding Self-Efficacy Scale. Subsequently, the women in the experimental group received education and counseling services using the teach-back method, based on the content of the prepared Breastfeeding Education Guide, which covered topics such as the structure of breast milk, the benefits of breast milk for both the baby and the mother, the timing of initiating breastfeeding, signs of hunger in the baby, pre-breastfeeding preparation, breastfeeding positions, and proper latch techniques. After the participants received the education, they were asked to reiterate what they had learned, and any misconceptions or misunderstandings were addressed through open-ended questions until clarity was achieved. This ensured accurate understanding and retention of the information. During the explanation phase, mothers were informed about breastfeeding and how to continue breastfeeding. At the same time, mothers were told what to pay attention to during the breastfeeding process.

During the evaluation phase, the mother was asked to explain the information taught. If the mother cannot disclose the information or expresses it incorrectly, it was assumed that the mother did not understand the information being taught.

In the repetition phase, unexplained or incorrectly expressed information was re-explained. During the training, care was taken to use plain language that the mother could understand and to give mothers enough time to ask any questions they had about breastfeeding. Additionally, the information was divided into small sections.

During the re-evaluation phase, open-ended questions were asked to the mothers until they were sure that they understood the information to be taught.

The “Tell What You Learned Observation Tool” was used to evaluate the health professional who provided training with the Tell What You Learned method by monitoring and evaluating his application of the method by another health professional in the clinic. Thanks to this tool, effective use of the method, observation of the skill levels of the staff related to the method and deficiencies were corrected.

During the education for the experimental group, the researcher’s implementation of the teach-back method was evaluated using the Teach-Back Observation Tool by a healthcare professional specialized in the field of Birth and Women’s Health. Mothers in the control group were given standard breastfeeding training and consultancy services, which are provided in all hospitals within the scope of mother-baby-friendly hospital criteria, using brochures on the benefits of breast milk and breastfeeding positions. At the end of the education sessions, the LATCH Breastfeeding Assessment and Evaluation Scale and the Postpartum Breastfeeding Self-Efficacy Scale were administered again.

**Fig. 2:**
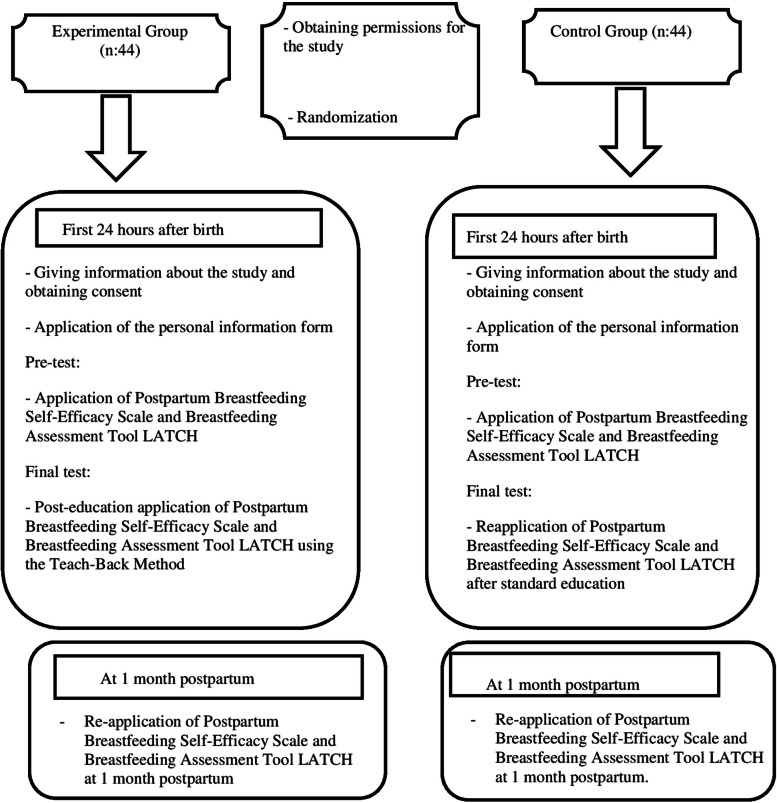
Study flow

A second interview was conducted during the 1-month postpartum check-up. During this interview, the scales were administered again to assess the retention of the acquired knowledge. The flow of the study is presented in Fig. 2.

### Study variables

#### Independent variables

The independent variables in this study are the breastfeeding education provided through the Teach-Back method, age, family type, employment status of the partner and the woman, educational background of the partner and the woman, history of receiving breastfeeding education, pregnancy and child number, and alcohol and cigarette use.

#### Dependent variables

The dependent variables of this research are the average scores of the postpartum breastfeeding self-efficacy scale and the LATCH assessment and Evaluation Scale.

### Statistical analysis

In the evaluation of the research findings, the SPSS (Statistical Package for the Social Sciences) version 25.0 (IBM Corp., Armonk, NY, USA) program was used for statistical analyses. Descriptive, graphical, and statistical methods were employed to examine whether the scores obtained from each continuous variable followed a normal distribution. The Kolmogorov-Smirnov test was used to assess the normality of the scores derived from a continuous variable using statistical methods. In addition to descriptive statistical methods, the independent samples t-test was used for comparisons between the two groups with normally distributed data, and the Mann-Whitney U test was used for the data that did not follow a normal distribution. For comparisons among more than two groups, the Kruskal-Wallis test was used, and the Bonferroni test was employed to determine the source of the difference between the groups. The Chi-square tests were used for qualitative comparisons between the groups. The Spearman correlation test was used to examine the level of relationship between two continuous variables. In repeated measures with multiple measurements, the Friedman test was used to test for differences, while the Wilcoxon test was used for repeated measures with two groups. The results were evaluated as statistically significant if *p* < 0.05 at a confidence interval of 95%.

## Results

### Socio-demographic characteristics of the participants

A total of 88 participants were included in the study, with 44 women in each group. The mean age of the experimental group mothers who received education using the Teach-Back method was calculated as 26.23 years (SD: 4.77). When examining the educational levels of the experimental group mothers, it was determined that 31.8% had primary education, 40.9% had secondary education, and 27.3% were university graduates. Regarding the educational levels of the participants’ spouses, it was found that 29.5% had primary education, 29.5% had secondary education, and 40.9% were university graduates. Furthermore, among the experimental group mothers who received education using the Teach-Back method, it was determined that 59.1% had income equal to their expenses, 18.2% used cigarettes, and 22.7% consumed alcohol. It was found that 45.5% of the mothers were experiencing their first pregnancy, 50% were having their first childbirth, 11.4% had previous abortions, 9.1% had experienced miscarriages, and 70.5% received breastfeeding education during pregnancy (Table 1).

**Table 1:** Socio-demographic characteristics of participants

		Experimental (*n* = 44)	Control(*n* = 44)	Significance	
Variables (*N* = 88)	Category	*n*(%)	*n*(%)	t/χ2	P
**age(year)**					
**mean(SD)**	All	26,23(4,77)	25,05(4,16)	1,238^a^	0,219
**Educational status**	Primary education	14(31,8)	14(31,8)	0,659^b^	0,719
	Secondary education	18(40,9)	21(47,7)		
	University	12(27,3)	9(20,5)		
**Partner’s educational status**	Primary education	13(29,5)	12(27,3)	1,346^b^	0,510
	Secondary education	13(29,5)	18(40,9)		
	University	18(40,9)	14(31,8)		
**Working status in an income generating job**	Yes	17(38,6)	13(29,5)	0,455^c^	0,500
	No	27(61,4)	31(70,5)		
**Income status**	Expenses less than income	10(22,7)	14(31,8)	0,917^b^	0,632
	Income equals expenses	26(59,1)	23(52,3)		
	Income greater than expenses	8(18,2)	7(15,9)		
**Smoking**	Yes	8(18,2)	5(11,4)	0,361^c^	0,548
	No	36(81,8)	39(88,6)		
**Alcohol use**	Yes	10(22,7)	9(20,5)	0,000^c^	0,999
	No	34 (77,3)	35 (79,5)		
**Number of pregnancies**	1	20(45,5)	16(36,4)	0,978^b^	0,613
	2	13(29,5)	17(38,6)		
	≥ 3	11(25,0)	11(25,0)		
**Number of births**	1	22(50,0)	19(43,2)	1,276^b^	0,528
	2 ≥ 3	13(29,5) 9(20,5)	18(40,9) 7 (15,9)		
**Number of miscarriages**	Yes	4(9,1)	6(13,6)	-^d^	0,739
	No	40(90,0)	38(86,4)		
**Number of abortions**	Yes	5(11,4)	4(9,1)	-^d^	0,999
	No	39(88,6)	40(90,9)		
**Obtaining information about breast milk and breastfeeding during pregnancy**	Yes	31(70,5)	26(59,1)	0,797^c^	0,372
	No	13(29,5)	18(40,9)		

*p* > 0.05; a(t): Independent Sample t-test; χ2 = Chi-Square Tests (b: Pearson Chi-Square, c: Yates Chi-Square Test, d: Fisher’s Exact Test); *SD *Standard deviation

The control group mothers who received standard education had a mean age of 25.05 years (SD: 4.16). It was determined that the average duration of marriage for the control group mothers who received standard education was 3.8 years (SD: 2.1). When examining the educational levels of the control group mothers who received standard education, it was found that 31.8% had primary education, 47.7% had secondary education, and 20.5% were university graduates. Regarding the educational levels of the participants’ spouses in the control group, it was determined that 27.3% had primary education, 40.9% had secondary education, and 31.8% were university graduates. Furthermore, among the control group mothers who received standard education, it was found that 52.3% had income equal to their expenses, 11.4% used cigarettes, 20.5% consumed alcohol, and 9.1% used medication regularly. It was determined that 36.4% of the mothers were experiencing their first pregnancy, 43.2% were having their first childbirth, 9.1% had previous abortions, 13.6% had experienced miscarriages, and 59.1% received breastfeeding education during pregnancy (Table 1).

No significant difference was found in the distribution of socio-demographic characteristics among the participants according to the study groups (*p* > 0.05) (Table 1).

### Postpartum Breastfeeding Self-Efficacy Scale scores

In the experimental group, consisting of mothers who received education through the Teach-Back method, it was determined that the mean pre-education, 24-hour postpartum and 1-month postpartum scores on the postpartum breastfeeding self-efficacy scale were 46.41 ± 11.26, 66.23 ± 6.94, and 67.84 ± 6.27, respectively. Significant differences were found in the mean scores of the postpartum breastfeeding self-efficacy scale within the study period (χ2 = 72.510; *p* < 0.001). Subgroup analyses revealed significant differences at all measurement times (Table 2).

**Table 2:** Comparison of postpartum breastfeeding self-efficacy scale scores by groups

Type of measurement	Measurement time	Experimental(*n* = 44)	Control(*n* = 44)	Significance	
		Mean ± SD	Mean ± SD	Z	*P*
**BSES**	**Pre-education**^**0**^	46,41 **±** 11,26	48,77 **±** 14,71	-1,298	0,194
	**24-Hour**^**1**^	66,23 **±** 6,94	50,86 **±** 13,98	**-6,287**	< 0,001*
	**1-month**^**12**^	67,84 **±** 6,27	49,70 **±** 16,47	**-7,182**	< 0,001*
**Significance**	**χ2**	**72,510**	**22,479**		
	**P**	< 0,001*	< 0,001*		
	Difference^**^	**0 < 1 < 2**	**0 < 1,2**		

*: *p* < 0.05; Z = Mann-Whitney U Test; χ2 = Friedman Test; **: Wilcoxon Signed-Ranks Test Mean: Mean, *SD *Standard Deviation

In the control group, which received standard education, it was determined that the mean pre-education, 24-hour postpartum and 1-month postpartum scores on the postpartum breastfeeding self-efficacy scale were 48.77 ± 14.71, 50.86 ± 13.98, and 49.70 ± 16.47, respectively. Significant differences were found in the mean scores of the postpartum breastfeeding self-efficacy scale in the control group during the study period (χ2 = 22.479; *p* < 0.001). The subgroup analyses revealed that the differences were between the pre-education scores and the scores obtained in the post-education measurements (Table 2).

In the pre-education measurements, there was no significant difference in the mean scores of the postpartum breastfeeding self-efficacy scale between the study groups (experimental and control) (*p* > 0.05). However, in the post-education measurements at 24 h and 1 month, it was found that the mean score of the postpartum breastfeeding self-efficacy scale in the experimental group was significantly higher than the mean score of the control group (Z=-6.287 and Z=-7.182; *p* < 0.001). From this finding, it was determined that the mothers who received education through the teach-back method had a higher level of breastfeeding self-efficacy (Table 2).

### Mean scores of LATCH Breastfeeding Assessment and Evaluation Scale

In the experimental group of mothers who received education through the Teach-Back method, it was found that the mean LATCH scores before education, at 24 h post-education, and at 1 month post-education were 7.73 ± 1.81, 8.66 ± 1.61, and 9.95 ± 0.30, respectively. During the follow-up period, there was a significant difference in LATCH score means in the experimental group (χ2 = 65,442; *p* < 0,001). The subgroup analyses revealed significant differences at all measurement time points (Table 3).

**Table 3:** Comparison of latch breastfeeding assessment and evaluation scale scores by groups

Type of measurement	Measurement time	Experimental(*n* = 44)	Control(*n* = 44)	Significance	
		Mean ± SD	Mean ± SD	Z	*P*
**LATCH**	**Pre-education**^**0**^	7,73 **±** 1,81	8,33 **±** 2,08	-1,939	0,052
	**24-Hour**^**1**^	8,66 **±** 1,61	8,55 **±** 1,72	-0,186	0,853
	**1-month**^**2**^	9,95 **±** 0,30	8,77 **±** 1,67	**-4,546**	< 0,001*
**Significance**	**χ2**	**65,442**	**14,207**		
	***P***	< 0,001*	0,001*		
	Difference^**^	**0 < 1 < 2**	**0 < 1,2**		

*:*p* < 0.05; Z = Mann-Whitney U Test; χ2 = Friedman Test; **: Wilcoxon Signed-Ranks Test; Mean: Mean, *SD *Standard Deviation

In the control group of mothers who received standard education, it was found that the mean LATCH scores before education, at 24 h post-education, and at 1 month post-education were 8.33 ± 2.08, 8.55 ± 1.72, and 8.77 ± 1.67, respectively. During the follow-up period, there was a significant difference in LATCH score means in the control group (χ2 = 14,207; *p* = 0,001). The subgroup analyses revealed that the difference was between the pre-education scores and the scores obtained from post-education measurements (Table 3).

No significant difference was found in the mean LATCH scores between the study groups before education and at 24 h post-education (*p* > 0.05). However, in the measurement taken at 1 month post-education, it was found that the mean LATCH score of the mothers in the experimental group was significantly higher compared to the mean score of the mothers in the control group (Z=-4,546; *p* < 0,001). This finding indicates that the mothers who received education through the teach-back method had higher breastfeeding success (Table 3).

### The relationship between breastfeeding self-efficacy and breastfeeding success

In the experimental group, consisting of mothers who received education through the Teach-Back method, a significant and positive correlation was found between the postpartum breastfeeding self-efficacy scale scores and the LATCH scores in the measurements taken before education and at 1 month post-education (before education: *r* = 0.518, *p* < 0.001; 1 month: *r* = 0.319, *p* = 0.035). These findings indicate that as breastfeeding self-efficacy levels increase in educated mothers, breastfeeding success also increases (Table 4).

**Table 4:** The relationship between breastfeeding self-efficacy and breastfeeding success among participants

	Experimental		Control	
	BSES	LATCH	BSES	LATCH
Measurement time	*R*	*p*	*r*	*p*
**Pre-Education**	0,508	< 0,001*	0,485	0,001*
**24-Hour**	0,154	0,319	0,514	< 0,001*
**1-month**	0,319	0,035*	0,708	< 0,001*

*p < 0,05, r = Spearman Correlation

In the control group, who received standard education, a significant and positive correlation was found between the postpartum breastfeeding self-efficacy scale scores and the LATCH scores in all measurements (before education: *r* = 0.485, *p* = 0.001; 24 h: *r* = 0.514, *p* < 0.001; 1 month: *r* = 0.708, *p* < 0.001). These findings indicate that as breastfeeding self-efficacy levels increase in mothers in the control group, breastfeeding success also increases (Table 4).

## Discussion

The aim of the study was to evaluate the impact of breastfeeding education using the Teach-Back method on mothers’ breastfeeding success and breastfeeding self-efficacy. Education and counseling are important for initiating and sustaining breastfeeding. Before the implementation of the Teach-Back method, there was no significant difference in breastfeeding self-efficacy levels between the groups. However, after the implementation of the Teach-Back method, a statistically significant difference in breastfeeding self-efficacy levels was observed in favor of the experimental group in the assessments conducted at 24 h and 1 month postpartum.

When comparing the mothers who received education through the Teach-Back method with the mothers who received standard education, it was found that the mothers who received education through the Teach-Back method had higher levels of breastfeeding self-efficacy. The significant improvement in self-efficacy levels at postpartum 24 h in the experimental group should be considered as an indicator of the success of the Teach-Back method. Furthermore, within-group evaluations showed an increase in breastfeeding self-efficacy levels at 1 month compared to the baseline level in both the experimental and control groups. However, while breastfeeding self-efficacy consistently increased throughout all measurement periods in the experimental group, the increase in the control group was inconsistent and observed between the baseline measurement and postpartum 24 h to 1 month periods. Similar to this study, Noel-Weiss et al. conducted a randomized controlled study investigating the impact of a prenatal breastfeeding workshop on mothers’ breastfeeding self-efficacy. They reported that in postpartum assessments conducted at 4 and 8 weeks after the workshop, breastfeeding self-efficacy was significantly higher in the intervention group [25]. According to Uçtu and Özerdoğan, the breastfeeding education program developed using the teach-back method was reported to increase breastfeeding self-efficacy in the experimental group [21]. The results of this research support the results of the abovementioned studies.

In the pre-training assessment, it was observed that as the educational level increased in both groups, mothers’ breastfeeding self-efficacy also increased. While this increase continued in the post-education period in the control group, it became statistically insignificant in the experimental group. This can be explained by the effect of the teach-back method. Studies in the literature indicate that as women’s education levels increase, breastfeeding self-efficacy also increases [26–29]. The findings of the control group and pre- education experimental group in the study support the positive relationship between education level and breastfeeding self-efficacy reported in the literature.

In the study, it was observed that in all evaluations conducted in the experimental group, and in the control group in the postpartum first month, the mothers with a partner who had a university degree had higher breastfeeding self-efficacy compared to those whose partner had completed primary education. This result can be associated with an increase in spousal support as the partner’s education level increases, leading to a higher sense of self-efficacy for the mother. This finding is supported by a study conducted by Şenol and Pekyiğit, which shows that as the partner’s education level increases, women’s breastfeeding self-efficacy levels also increase [30]. In another study by Maleki Saghooni et al., breastfeeding self-efficacy rates were found to be higher in those with more social support. The study results support the literatüre [17].

In the study, there was no significant difference in breastfeeding success between the experimental group who received breastfeeding education using the teach-back method and the control group who received standard education during the pre-education period and at postpartum 24 h evaluation. However, in the postpartum 1-month evaluation, higher breastfeeding success was observed in the experimental group. This finding is consistent with the research conducted by Beake et al., where they examined the relationship between structured breastfeeding education programs and standard breastfeeding education programs and found that structured breastfeeding education programs were more effective in achieving breastfeeding success compared to standard education programs [31]. In another study, it was found that a postpartum education program based on the teach-back method resulted in approximately a twofold increase and improvement in women’s quality of life compared to the standard care program [32]. The findings of our study support the literature results indicating that structured breastfeeding programs are more effective in improving breastfeeding success compared to standard education programs.

In our study, sociodemographic variables associated with the mother’s LATCH breastfeeding assessment and evaluation scale scores were found to be the mother’s education level and smoking status in the control group, and both the mother’s and partner’s education level, the mother’s active employment status, and the mother’s previous breastfeeding education status in both the experimental and control groups. The study revealed that the mothers, who had higher education levels, were actively employed, previously received breastfeeding education, and were non-smokers had better breastfeeding success. This finding is consistent with the literature, which reports that socio-demographic factors influence a mother’s breastfeeding success [33–36]. The study found that increased self-efficacy was associated with improved breastfeeding success in both study groups. This result can be attributed to the higher breastfeeding desire among women as their self-efficacy perception increases, leading to fewer breastfeeding problems. A study conducted in 2022 reported a positive and significant relationship between breastfeeding success scores and breastfeeding self-efficacy scores [37]. The findings of that study are similar to the results of this study.

## Limitations of the study

This study was conducted only with the postpartum women in the specified hospital; therefore, the data are limited to the participants from that hospital and cannot be generalized to all postpartum women.

### Strengths of the research

- Data collection by the researcher through face-to-face interviews before and immediately after the training.
- Providing monitoring and support in initiating breastfeeding by having the first interview (on the first postpartum day) in the obstetrics and gynecology ward while the woman is still in the hospital.
- Providing more effective participation as women need education in the early postpartum period.
- The research is a randomized controlled study,
- The sample size was determined by power analysis,
- Use of standard measuring tools.
- The strength of the study is that the woman’s social supporters (mother, wife, etc.) are present and receive counseling when she starts breastfeeding.

### Weaknesses of the research

- In the study carried out during the return to normal period after the pandemic, time losses occurred due to reasons such as women not wanting to spare time during their 1st month check-ups.
- Data loss occurred in the study because some women could not be reached.

## Conclusion

This research revealed that the mothers who received education through the “teach-back” method had higher levels of breastfeeding self-efficacy and breastfeeding success. Furthermore, it was found that as breastfeeding self-efficacy increased among the mothers who received the teach-back method education, breastfeeding success also increased. The results of the study have significant implications for clinical practice in terms of demonstrating the effectiveness of breastfeeding education through the teach-back method in enhancing breastfeeding self-efficacy and breastfeeding success in a short period of time, ensuring the sustainability of the education, and guiding other related studies. It is recommended to integrate the teach-back method into routine breastfeeding education and incorporate it into all healthcare education and counseling services, and further large sample studies should be conducted to examine its effectiveness.

## Data Availability

The data that support the findings of this study are available from Eda Cangöl but restrictions apply to the availability of these data, which were used under license for the current study, and so are not publicly available. Data are how ever available from the authors upon reasonable request and with permission of Eda Cangöl.
